# Cytotoxicity of a New Nano Zinc-Oxide Eugenol Sealer on Murine Fibroblasts 

**DOI:** 10.7508/iej.2015.04.004

**Published:** 2015

**Authors:** Maryam Javidi, Mina Zarei, Salma Omidi, Ahmad Ghorbani, Maryam Gharechahi, Maryam Shayani Rad

**Affiliations:** a *Dental Material Research Center, Department of Endodontics, Dental School, Mashhad University of Medical Sciences, Mashhad, Iran; *; b * Department of Endodontics, Dental School, Mazandaran University of Medical Sciences, Sari, Iran;****c***; c *Pharmacological Research Center of Medicinal Plants, Medical School, Mashhad University of Medical Sciences, Mashhad, Iran;*; d * Student Research Committee (SRC), Mashhad University of Medical Sciences, Mashhad, Iran*

**Keywords:** Cytotoxicity, MTT Assay, Nanoparticles, Root Canal Sealer, Zinc-Oxide Eugenol

## Abstract

**Introduction:**

The aim of this study was to evaluate the cytotoxicity of a new nano zinc-oxide eugenol (NZOE) sealer in comparison with AH-26 and Pulpdent root canal sealers.

**Methods and Materials:**

The L929 mouse fibroblast cells were cultivated and incubated for 24, 48 or 72 h with different dilutions (1/1, 1/2, 1/4, 1/8, 1/16 and 1/32) of culture media previously exposed to either of the test sealers naming NZOE, AH-26 or Pulpdent. At the end of incubation period, the effect of sealers on cell viability was evaluated using Mosmann’s Tetrazolium Toxicity (MTT) colorimetric assay. The data was compared using the one-way analysis of variance (ANOVA) followed by the Tukey’s post hoc test for multiple comparisons.

**Results:**

After 24, 48 or 72 h, both NZOE and Pulpdent sealers inhibited cell viability at 1/1, 1/2 and 1/8 dilutions. Within the 24 and 48 h, the AH-26 sealer reduced the cell viability at all dilutions except the 1/32 solution; however after 72 h even the 1/32 dilution was cytotoxic.

**Conclusion:**

The biocompatibility of the nano zinc-oxide eugenol sealer was comparable to Pulpdent sealer and lower than AH-26.

## Introduction

Use of endodontic sealers with ideal properties is necessary for the success of root canal treatment [1]. An ideal sealer should be biologically compatible and well tolerated by periradicular tissues [2]. Unfortunately, it is difficult to produce sealers with proper physicochemical properties and biological compatibility. Materials that are well tolerated by tissues compromise sealer properties, and *vice versa* [3]. Zinc-oxide eugenol (ZOE)-based sealers are one of the most common and conventional sealers used in endodontic treatment [4]. These sealers have undergone a lot of modifications and different commercial products of ZOE-based sealers are available. 

At present, nano-technology is used to produce a large number of dental materials, including light-cured restorative composite resins and their bonding systems, impression materials, ceramics, dental implant covering layers and fluoride mouthwashes [5, 6]. Other advantages of nanoparticles, which have attracted attention in endodontics, are their better penetration into the dental tubules, profound antibacterial properties and decreased microleakage [6-10]. Because of these favorable properties, utilization of nanoparticles in production of endodontic sealers has become the center of interest, recently [11]. Several researchers incorporated quaternized polyethylenimine nanoparticles or chitosan nanoparticles into different sealers and evaluated their biocompatibility, antibacterial and physiochemical properties [12-17]. Sousa *et al.* [18] synthesized and characterized ZOE nanocrystals and evaluated their biological properties for application in dentistry, particularly in endodontics. 

Recently, a new endodontic sealer with nano-sized ZOE powder particles (NZOE) has been developed in the Dental Material Research Center, Mashhad University of Medical Sciences, Mashhad, Iran. This sealer is similar to various ZOE-based sealers, but with different sizes of ZOE nanoparticles [19].

When a new dental material is introduced, its biocompatibility should be determined. Any nano endodontic sealer must remain compatible with periapical tissues during long-time contact [14]. 

**Figure 1:**
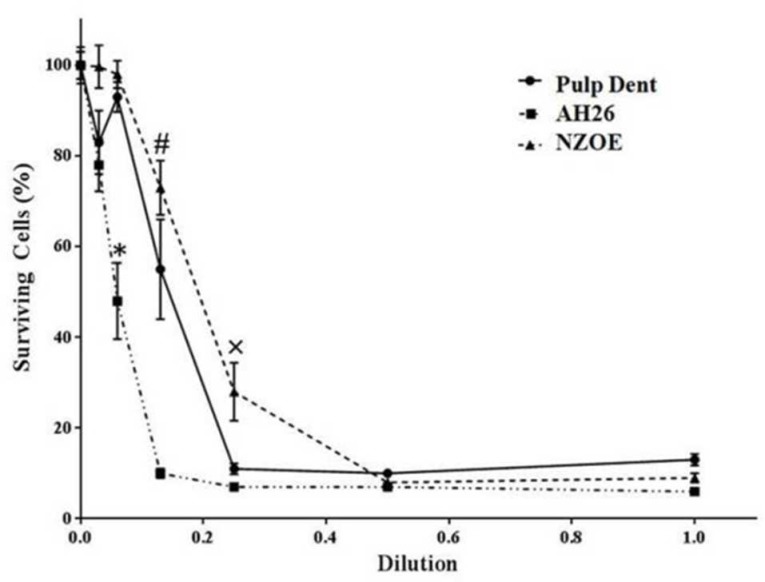
Effects of AH-26, Pulpdent and NZOE sealers on viability of L929 mouse fibroblasts after 24 h

Therefore, several biocompatibility tests including cytotoxicity, intraosseous implantations and subcutaneous implantations have been proposed [20]. The aim of this study was to evaluate the cytotoxicity of NZOE sealer in comparison with AH-26 and Pulpdent root canal sealers.

## Materials and Methods

AH-26 sealer (Dentsply, De Trey, Konstanz, Germany) and Pulpdent sealer (Pulpdent, Watertown, MA, USA) were purchased. Dimethyl sulfoxide (DMSO), penicillin-streptomycin and 3-(4, 5-Dimethyl-2-thiazolyl)-2, 5-Diphenyl-2H-tetrazolium bromide (MTT) were obtained from Sigma (Sigma Chemical Co., St. Louis, Missouri, USA). Also the Dulbecco’s Modified Eagle’s Medium (DMEM) and fetal bovine serum (FBS) were bought from Gibco (Gibco Chemical Co., Carlsbad, CA, USA).

***Preparation of nano zinc-oxide Eugenol (NZOE) sealer***

NZOE was prepared *via* a sol-gel method as described in our previous work [7]. Briefly, a solution of gelatin was prepared by dissolving 10 g gelatin in 150 mL deionized water at 60^°^C. Then, appropriate amounts of zinc-nitrate [Zn(NO_3_)_2_.6H_2_O] was dissolved in a minimum volume of deionized water at room temperature. The two prepared solutions were mixed and stirred for 8 h while the temperature was kept at 80^°^C. Finally, the prepared resin was dried at 500^°^C, in which the pure NZOE powder was obtained.

***Preparation of sealer extract***

The NZOE sealer was sterilized under UV light for 24 h. Then, all the test sealers were prepared according to the user’s manuals and immediately inserted in a 24-well plate before setting (2 wells for each sealer). After that, 2.5 mL DMEM was added to each well and the plate was incubated in the dark for 24 h at 37^°^C. After incubation, these original extracts (1/1 dilution) were passed through 0.22 μm filters and then serially diluted in fresh DMEM supplemented with antibiotic and 10% FBS. Different dilutions (1/1, 1/2, 1/4, 1/8, 1/16 and 1/32) of each sealer were used for cytotoxicity assay.

***Cell culture and treatment***

L929 mouse fibroblast cells were cultivated in high-glucose DMEM supplemented with 10% FBS and penicillin (100 units/mL) and streptomycin (100 μg/mL) at 37^°^C in an atmosphere including 5% CO_2_. Trypsin was used to passage cultures whenever they were grown to confluence. The cells at sub-confluent stage were harvested from culture flask and after checking the cell viability using trypan blue exclusion technique, they were seeded overnight in a 96-well culture plate. Then, to test cytotoxicity of sealers, the culture media was exchanged with fresh one containing varying dilutions (1/1 to 1/32) of each sealer. Three wells were allocated for each dilution of sealers, and the experiment was repeated three times (*n*=9). Then, the cells were further incubated for 24, 48 or 72 h and observed under light inverted microscope for shape, granulation and anchorage independency [21, 22]. Untreated cells were considered as negative control.

***MTT cell viability assay***

At the end of incubation, the MTT solution (3-{4,5-dimethylthiazol-2-yl}-2,5-diphenyl tetrazolium bromide) in phosphate-buffered saline (5 mg/mL) was added to each well of culture plate to make final concentration of 0.5 mg/mL and the cells were incubated for 2 h. Then, the supernatant was removed and the resulting formazan was dissolved by adding 200 μL DMSO to each well. The optical density of formazan dye was read at 545 nm against 620 nm as back ground by Elisa reader (Awareness Technology Inc). The percentage of viable cells in each well was calculated relative to control cells set to 100% [23, 24]. Also the IC50 value (Concentration/dilution at which 50% inhibition of cell proliferation was created) was evaluated.

***Statistical tests***

Data normality was tested with the Kolmogorov-Smirnov test. The results were compared using the one-way analysis of variance (ANOVA) followed by the Tukey’s post hoc test for multiple comparisons. The level of significance was set at 0.05.

## Results

***Cell viability after 24 h***

Pulpdent sealer at dilutions of 1/8, 1/16 and 1/32 had no significant effect on the viability of L929 cells (Figure 1). However at 1/1, 1/2 and 1/4 dilutions, it decreased cell surviving from 100±3% (control) to 13±1% (*P*<0.001), 10±0.8% (*P*<0.001) and 11±1% (*P*<0.001), respectively. Also, the percent of viable cells at presence of 1/1, 1/2 and 1/4 dilutions of NZOE was 9±1% (*P*<0.001), 7±0.8% (*P*<0.001), and 28±6% (*P*<0.001), respectively. Regarding AH-26, in addition to 1/1, 1/2 and 1/4 dilutions, cytotoxicity was also observed at 1/8 dilution of (10±1%, *P*<0.001) (Figure 1).

**Figure 2:**
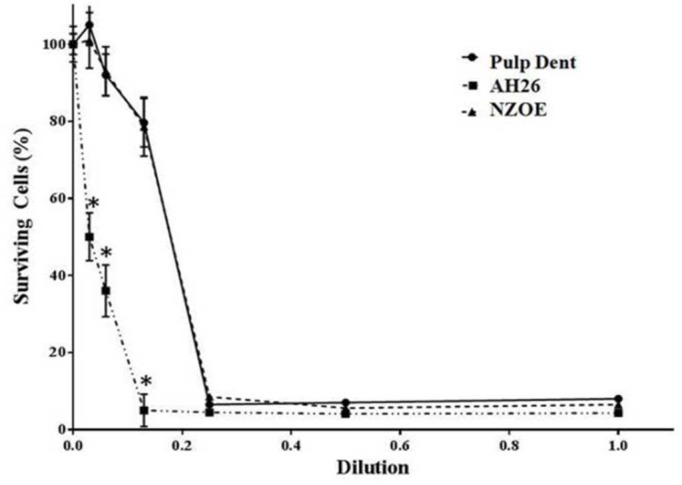
Effects of AH-26, Pulpdent and NZOE sealers on viability of L929 mouse fibroblasts after 48 h

Regarding the NZOE sealer, cytotoxicity at dilutions of 1/4 and 1/8 was lower than that of Pulpdent (*P*<0.05) and AH-26 sealers (*P*<0.001). The IC50 value for Pulpdent, AH-26 and NZOE was found to be at dilutions of 0.13, 0.05 and 0.19, respectively.

***Cell viability after 48 h***

Pulpdent and NZOE sealers exhibited no cytotoxicity at dilutions of 1/8, 1/16 and 1/32 (Figure 2). However, exposure of the cells to dilutions of 1/1, 1/2 and 1/4 of these sealers significantly decreased the cell viability from 100±4.6% (control) to 8±0.7%, 7±0.6% and 6±0.4% for Pulpdent and 6±0.2%, 5±0.2% and 8±0.3% for NZOE, respectively (*P*<0.001). The cells incubated with dilution of 1/8 of AH-26 similar to dilutions of 1/1, 1/2 and 1/4 showed a significant decrease in their viability (5±0.3%) in comparison with the positive control samples (*P*<0.001).

Statistical analysis showed that cytotoxicity of NZOE at dilutions of 1/8, 1/16 and 1/32 was lower than that of AH-26 sealer (*P*<0.001). After a 48-h incubation period, the IC50 value for Pulpdent, AH-26, and NZOE was observed at dilutions of 0.16, 0.02 and 0.16, respectively (Figure 2).

***Cell*** ***viability*** ***after 72 h***

**Figure 3:**
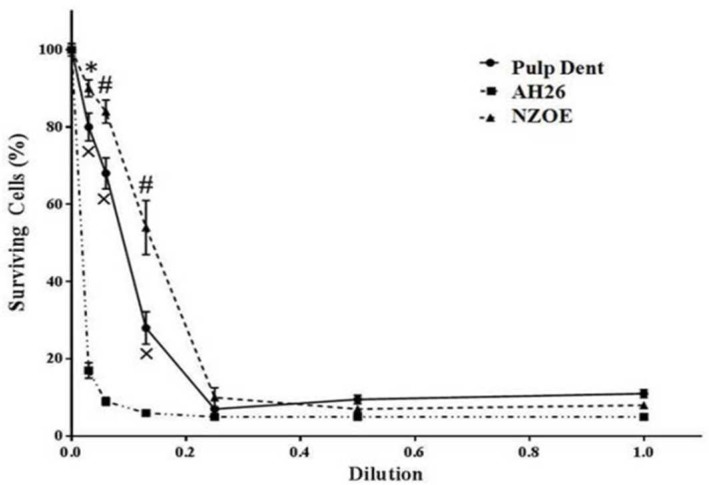
Effects of AH-26, Pulpdent and NZOE sealers on viability of L929 mouse fibroblasts after 72 h

As shown in Figure 3, Pulpdent sealer at dilutions of 1/16 and 1/32 has no significant effect on cell viability. However at dilutions of 1/1, 1/2, 1/4 and 1/8, it decreased the cell survival from 100±1.6% (control) to 10±1% (*P*<0.001), 9±1% (*P*<0.001), 7±0.3% (*P*<0.001) and 28±4% (*P*<0.001), respectively. The percent of viable cells in 1/1, 1/2, 1/4 and 1/8 dilutions of NZOE was 8±0.6% (*P*<0.001), 6±0.5% (*P*<0.001), 1±2.5% (*P*<0.001) and 54±7% (*P*<0.001), respectively. On the other hand, AH-26 was cytotoxic at all dilutions of 1/1, 1/2, 1/4, 1/8, 1/16 and 1/32 so that the level of cell surviving significantly (*P*<0.001) decreased to 4.8±0.2, 4.6±0.3, 4.7±0.3, 5.8±0.5, 9±1 and 17±2 percent, respectively.

Cytotoxicity of AH-26 at dilutions of 1/8, 1/16 and 1/32 was higher than that of Pulpdent (*P*<0.001) and AH-26 (*P*<0.001). The IC50 value for Pulpdent, AH-26 and NZOE was found to be at dilutions of 0.07, 0.006 and 0.12, respectively (Figure 3).

## Discussion

The aim of the present study was to evaluate the cytotoxicity of a newly introduced NZOE sealer in comparison with AH-26 and Pulpdent endodontic sealers. The AH-26 is a popular and commonly used epoxy resin sealer with established toxic properties especially during the first 24 h [4]. The Pulpdent is also a commercially available ZOE-based sealer. 

To evaluate the cytotoxicity of endodontic materials, in some studies the materials have been placed in direct contact with cells [25-27], while in some other works, the extract of sealers has been mixed with the cell culture media [28-34]. Direct placement of the sealer in the culture plate may result in physical injuries to cells and increases the risk of bacterial contamination. Therefore, in the present study the second technique, *i.e*. the sealer extract technique, was used. Since this was the first study that evaluated the cytotoxicity of NZOE sealer, different dilutions of the sealer extract were prepared and used similar to the study by Bin *et al.* [32]. 

In the clinical settings, the sealer is immediately placed within the root canal after being mixed. If the sealer comes into contact with periapical tissues, the maximum toxic effect of the sealer occurs before its setting. In the present *in vitro* study, an attempt was made to simulate the maximum cytotoxic effect of the sealer in the human body. Therefore, the sealers were added to culture media 5 min after mixing and the culture media was placed in contact with the sealer for 24 h to ensure the transfer of all the toxic materials of the sealer into the culture media.

Our results showed that all the three sealers were highly cytotoxic at 1/1, 1/2 and 1/4 dilutions since they had not been diluted (1/1) or were diluted minimally (1/2 and 1/4). These dilutions of sealers resulted in about 90% cellular death during the first 24 h. Therefore, more cytotoxicity could not be expected after 48 and 72 h with similar dilutions. However, all sealers at dilutions of 1/8, 1/16 and 1/32 exhibited higher cytotoxic effect after 72 h compared to 24 h incubation. An increase in the cytotoxicity of sealers with time is similar to the results observed in studies by Karapinar *et al.* [31] and Bouillarge *et al.* [35].

Among all three sealers at the three time intervals, AH-26 had the highest cytotoxic effect, followed in descending order by Pulpdent and NZOE sealers. In a study by Badol *et al.* [33], AH-26 showed severe toxicity which became mild after one month while Pulpdent sealer showed severe to moderate toxicity. Until now no study has evaluated the toxic effect of NZOE sealer. However, Sousa *et al.* [18] evaluated the biological properties of ZOE nanocrystals through intra-osseous implantation and reported that the nanocrystals are biocompatible, well tolerated and allow bone formation and remodeling. Several researchers evaluated the biocompatibility of other nanoparticles. Gomes *et al.* [5] evaluated the tissue response after irrigation with silver nanoparticles and concluded that these particles are biocompatible, especially at low concentrations. Dianat *et al.* [36] showed that the cytotoxicity of CH nanoparticles was similar to that of conventional CH. Abramovitz *et al.* [11] revealed that incorporation of 1% quaternized polyethylenimine (QPEI) nanoparticles into the sealers, did not impair their biocompatibility. 

Shantiaee *et al.* [37] compared the cytotoxicity of nano-silver-coated gutta-percha with Guttaflow and normal gutta-percha on L929 fibroblasts with MTT assay after 1 h; nano-silver-coated gutta-percha and Guttaflow had the highest and the lowest cytotoxicity, respectively. After 24 h and 1 week, no significant differences were observed. Barros *et al.* [14] concluded that the incorporation of 2% QPEI nanoparticles into AH-Plus and Pulp Canal Sealer (PCS), modulates the proliferation and differentiation of bone cells, depending on the sealer and the cell type, without increasing the sealer cytotoxicity.

Our results are consistent with those of Bae *et al.* [38] who showed that in the MTT test the cytotoxic effect of a ZOE-based sealer at 1/2, 1/4 and 1/16 dilutions was less than that of AH-26 sealer. In addition, in a study by Huang *et al.* [29] the cytotoxic effects of AH-26 sealer at 1/2, 1/4 and 1/8 dilutions were greater than that of ZOE-based sealer. 

In the majority of *in vitro* cytotoxicity studies, the toxic effects of epoxy-resin sealers were high, especially shortly after mixing [20, 28, 29, 32, 35, 39]. In addition, the cytotoxic effects of ZOE-based sealers were similar, but the toxic effects were lower than that of AH-26 sealer [29, 33, 35]. Huag *et al.* [28] reported that the cytotoxicity of AH-26 and AH-Plus sealers on days 1, 2 and 3 are higher than ZOE-based sealers.

## Conclusion

In conclusion, the cytotoxicity of the tested nano-sealer was comparable to that of Pulpdent and was lower than AH-26 sealer. Further studies on the possible use of NZOE sealer as a new root canal filling material seems necessary.
